# Global epidemiology of amyloid light-chain amyloidosis

**DOI:** 10.1186/s13023-022-02414-6

**Published:** 2022-07-19

**Authors:** Nishant Kumar, Nicole J. Zhang, Dasha Cherepanov, Dorothy Romanus, Michael Hughes, Douglas V. Faller

**Affiliations:** 1 RwHealth, Level 39, One Canada Square, Canary Wharf, London, E14 5AB GB UK; 2 Takeda Development Center Americas, Inc., 95 Hayden Ave., Lexington, MA 02421 USA

**Keywords:** Amyloid light-chain amyloidosis, Prevalence, Incidence, Epidemiology, Global, Rare disease

## Abstract

**Background:**

Amyloid light-chain (AL) amyloidosis is an ultra-rare disease associated with significant morbidity and mortality. Few studies have examined the global epidemiology of this condition.

**Methods:**

This study estimated the diagnosed incidence and 1-year, 5-year, 10-year, and 20-year period prevalence of AL amyloidosis in 2018 for countries in and near Europe, and in the United States (US), Canada, Brazil, Japan, South Korea, Taiwan, and Russia. A systematic literature review (SLR) was conducted to identify country-specific, age- and gender-specific diagnosed incidence of AL amyloidosis and observed survival data-point inputs for an incidence-to-prevalence model. Extrapolations were used to estimate incidence and prevalence for countries without registry or published epidemiological data.

**Results:**

Of 171 publications identified in the SLR, 10 records met the criteria for data extraction, and two records were included in the final incidence-to-prevalence model. In 2018, an estimated 74,000 AL amyloidosis cases worldwide were diagnosed during the preceding 20 years. The estimated incidence and 20-year prevalence rates were 10 and 51 cases per million population, respectively.

**Conclusions:**

Orphan medicinal product designation criteria of the European Medicines Agency or Electronic Code of Federal Regulations indicate that a disease must not affect > 5 in 10,000 people across the European Union or affect < 200,000 people in the US. This study provides up-to-date epidemiological patterns of AL amyloidosis, which is vital for understanding the burden of the disease, increasing awareness, and to further research and treatment options.

## Background

Amyloid light-chain (AL) amyloidosis is a rare multi-systemic, incurable protein misfolding disorder characterized by plasma cell dyscrasia and abnormal clonal plasma cell proliferation. AL amyloidosis impedes organ function, resulting in multi-organ dysfunction, organ failure, and in 24–37% of patients, death within 6 months of diagnosis [1, 2]. The majority of patients with AL amyloidosis are over 65 years old, and the mean age at diagnosis is 63 years [3].

Clinical manifestations of systemic AL amyloidosis resemble several common conditions found in the elderly; therefore, diagnosis often occurs at an advanced stage of disease after the onset of involvement and damage to critical organs, when treatment is largely ineffective [4–6]. The current staging system for AL amyloidosis is based on levels of circulating markers of cardiac and renal damage. Biomarkers of cardiac dysfunction are included in the Mayo 2004 staging system, which uses measurements of troponin T (cTnT) or TnI and N-terminal pro-brain natriuretic peptide (NT-proBNP) [7]. European modifications of this system use different thresholds of NT-ProBNP (2015 European modification) [8] and consider systolic blood pressure (2013 European modification) [9]. The Mayo 2012 staging system uses measurements of cTnT, NT-proBNP and the difference between involved and uninvolved free light chains (FLC-diff) [10]. The Boston University (BU) biomarker score uses BNP and TnI [11]. Renal staging in AL amyloidosis predicts risk of progression to dialysis based on estimated glomerular filtration rate (eGFR) and proteinuria [8]. Median overall survival for patients with Mayo (2012) Stage 1, 2, 3, and 4 AL amyloidosis is 94.1 months, 40.3 months, 14 months, and 5.8 months, respectively [10]*.*

Current therapy aims to control AL amyloidosis-related multi-organ dysfunction and improve patient survival [12]. Evaluating patients’ eligibility for stem cell transplantation is a first step in the treatment pathway. Daratumumab-based regimens represent emerging treatment options for newly diagnosed patients and those with relapse refractor disease [6, 13]. In clinical trials, the addition of daratumumab to the standard of care resulted in deeper and more rapid hematologic responses, improved organ responses and clinical outcomes, and an acceptable safety profile [14, 15]. These data resulted in accelerated United States (US) Food and Drug Administration (FDA) approval of subcutaneous daratumumab plus hyaluronidase in January of 2021 as the first and only agent specifically for the treatment of AL amyloidosis [16]. Ixazomib is an oral proteasome inhibitor (PI) currently approved for the treatment of multiple myeloma. A Phase 3 Tourmaline-AL1 trial (TOURMALINE-AL1 NCT01659658) evaluated ixazomib plus dexamethasone, for the treatment of patients with relapsed/refractory primary systemic AL Amyloidosis (RRAL), who had received one or two prior therapies. At interim analysis, treatment with ixazomib-dexamethasone was well tolerated, best hematologic response rate was 53% with ixazomib-dexamethasone vs. 51% with physician's choice of therapy (dexamethasone alone or with melphalan, cyclophosphamide, thalidomide, or lenalidomide), complete response rate was 26% vs. 18%, the median time to vital organ deterioration or mortality was 34.8 months vs. 26.1 months, and the median treatment duration was 11.7 months vs. 5.0 months. Although the primary endpoint of superior hematologic response rate was not met, all time-to-event endpoint data favored ixazomib-dexamethasone [17]. In a subgroup analysis of overall response rate (ORR) by prior PI exposure in Tourmaline-AL1, ORR was higher with ixazomib-dexamethasone compared to physician’s choice in PI-naïve patients than in PI-exposed patients, although the differences were not statistically significant [14, 18].

AL amyloidosis poses a substantial burden on the healthcare system in the US. In one US study, commercial and Medicare claims data were used to examine healthcare utilization and costs associated with AL amyloidosis between 2007 and 2015. Findings showed that rates of hospitalization among patients with AL amyloidosis declined from 57% in 2007 to 46% in 2015; however, hospitalizations were common, with half of the patients admitted at least once [3]. Mean total annual all-cause healthcare costs for patients with AL amyloidosis were $101,855, increasing from $92,513 in 2007 to $114,030 in 2015 [19].

To understand the true burden of AL amyloidosis, increase awareness, inform treatment options, and allow effective healthcare provision, it is essential to determine how many cases of AL amyloidosis exist globally. Few studies have examined the epidemiology of AL amyloidosis, and country-specific registries for AL amyloidosis are limited. The objective of this study was to examine the global epidemiology of AL amyloidosis, reporting annual incidence and period prevalence.

## Methods

### Study design

This study estimated the diagnosed incidence and (1-year, 5-year, 10-year, and 20-year) period prevalence of AL amyloidosis in 2018 in 31 countries in Europe (Austria, Belgium, Bulgaria, Croatia, Cyprus, Czech Republic, Denmark, Estonia, Finland, France, Germany, Greece, Hungary, Iceland, Republic of Ireland, Italy, Latvia, Liechtenstein, Lithuania, Luxembourg, Malta, Netherlands, Norway, Poland, Portugal, Romania, Slovakia, Slovenia, Spain, Sweden, United Kingdom), Brazil, Canada, Japan, Russia, South Korea, Taiwan and the US. An SLR was conducted to identify country-specific, age- and gender-specific diagnosed incidence rates and observed survival data-point inputs for an incidence-to-prevalence model.

### SLR

The SLR was performed according to the Preferred Reporting Items for Systematic Reviews and Meta-Analyses (PRISMA) guidelines [20]. The MEDLINE database was searched to identify records on AL amyloidosis published between January 1, 2005 and December 15, 2018. Search terms included: “immunoglobulin light chain amyloidosis” OR "AL amyloidosis" AND “population-based” OR “hospital-based” OR “clinic” OR “register*” OR “epidemiology*”. There was no eligibility requirement for adequate amyloid typing. No language limits were applied. A manual search was used to identify additional records in the gray literature or conference proceedings.

Inclusion criteria were (1) observational and non-interventional design; (2) with a clear definition of AL amyloidosis; (3) reporting incidence, prevalence, survival, or excess mortality of AL amyloidosis; (4) with estimates that could be considered representative and generalizable to their respective national populations. Records were excluded if they were reviews, opinion pieces, controlled trials, case reports, or if they were not population-based in design.

**Fig. 1:**
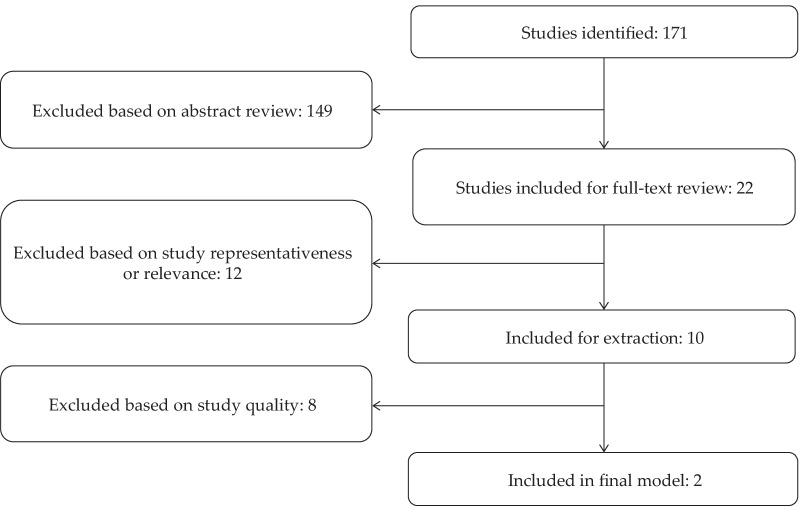
PRISMA diagram of identified and included records. *PRISMA* Preferred Reporting Items for Systematic Reviews and Meta-Analyses

Abstracts of all relevant records were reviewed for eligibility. A total of 171 records were identified, of which 10 were included for data extraction (Fig. 1) [3, 21–29]. There were 2 records each from France, Sweden, and the US, and one record each from Argentina, China, England, and Korea. Two records were included in the final incidence-to-prevalence model [3, 29].

### Analyses

#### Incidence of AL amyloidosis

Three population-based studies reporting directly measured estimates of incidence were identified in the SLR. Estimates included a crude incidence of 3 cases per million population (PMP) in England in 2008 [27], 11 cases per million-person years in Argentina from 2006 to 2014 [21], and a range of 9.7–14.0 cases per million-person years in the US from 2007 to 2015 [3] (Table 1).

**Table 1:** Incidence of AL amyloidosis reported in identified studies

Study	Country	Study period	Sex	Age (years)	Number of cases	Crude incidence (95% CI)	Incidence adjusted for age and sex^a^ (95% CI)
Aguirre et al. [21]	Argentina	2006–2014	Both	> 17	11	10.85 (6.16–19.11)^a^	6.13 (2.57–9.7)^c^
			Female	> 17	Not reported	7.51 (3.12–18.04)^a^	Not reported
			Male	> 17	Not reported	15.94 (7.6–33.44)^a^	Not reported
Pinney et al. [27]	England	2000–2008	Both	≥ 30	174	3^b^	Not reported
Quock et al. [3]	United States	2007–2015	Both	≥ 18	2207	9.7–14.0^a^	10.8–15.2
			Female	≥ 18	1020	8.3–11.9^a^	8.7–13.3
			Male	≥ 18	1187	10.7–16.4^a^	12.5–17.8

*CI* confidence interval

^a^Per million person-years

^b^Per million

^c^Adjusted for City of Buenos Aires, Argentina Census 2010

**Fig. 2:**
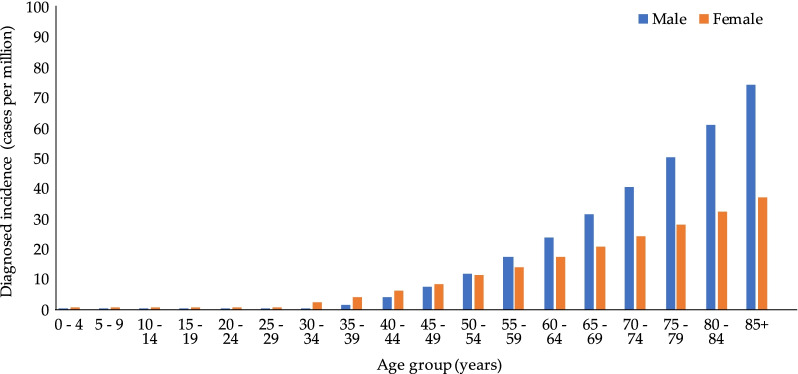
Age-specific incidence of AL amyloidosis, by sex. *AL* amyloid light-chain

Given the limited data available for incidence, age-specific incidence rates for the US were extracted [3] and applied to all countries under study. The study from England [27] was excluded as it ascertained cases by death certificates at the National Amyloid Centre (NAC) on the assumption that all cases with amyloidosis in England would be seen at the NAC, which is likely an underestimation. The study from Argentina [21] was excluded due to much smaller sample size compared to the US study [3] and incidence was determined based on 11 cases, compared to over 2000 cases in the US study. Little variation in annual incidence of AL amyloidosis between 2008 and 2015 was observed in the US study and by others [30]; therefore, an average incidence rate was determined for the following age groups: 18–34, 35–54, 55–64, and 65 + years. As the incidence of AL amyloidosis increased with age, a curve was fitted to the age group-specific incidence rates to facilitate interpolation of more granular age-specific incidence rates in 5-year groupings (Fig. 2). Age- and gender-specific incidence rates were applied to the corresponding population estimates for each country under study to derive incident cases and incidence. Incidence estimates were reported as PMP, calculated by dividing country-, age-, and gender-specific cases by United Nations’ country-, age-, and gender-specific population estimates [31].

#### AL amyloidosis survival

The countries under study were assumed to have well developed healthcare systems, such that access to and quality of healthcare was assumed not to vary sufficiently enough to cause substantial differences in AL amyloidosis survival. Therefore, data from a population-based study identified in the SLR reporting OS of AL amyloidosis in Sweden across four time periods of 1995–1999, 2000–2004, 2005–2009, and 2010–2013 was applied to all countries [29]. Using the mid-point of each of the four time periods, and the corresponding background survival estimates (defined as the proportion of observed survivors in the general population) for Sweden obtained from United Nations World Population Prospects 2017, we derived estimates of relative survival of AL amyloidosis (defined as the ratio of observed survivors among patients with AL amyloidosis [29] to expected survivors in the general population [31] for each year for years 1 through 20). Improvements observed in relative survival over the four time periods were incorporated into the analysis to estimate prevalence in 2018, which was used as the base year.

#### Prevalence of AL amyloidosis

**Fig. 3:**
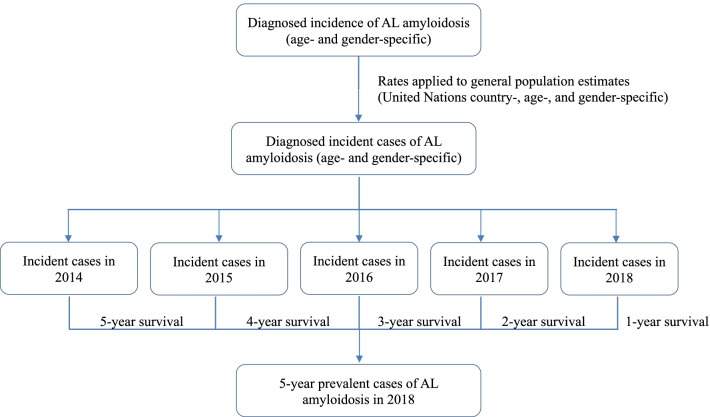
Estimating 5-year prevalent cases of AL amyloidosis in 2018. *AL* amyloid light-chain

Using the identified and derived estimated annual incidence and survival of AL amyloidosis, the period prevalence of AL amyloidosis was calculated for each country under study. Specifically, for 20-year prevalence, to estimate diagnosed prevalent cases of AL amyloidosis, the number of diagnosed incident cases each year for the preceding 20 years was estimated by multiplying the annual age- and gender-specific incidence estimates by the United Nations’ age-specific population estimates for each year and country from 1999 to 2018 [31]. Using annualized survival estimates for each year within 20 years following diagnosis, the number of prevalent cases that survived to 2018 were estimated cumulatively. Additionally, 1-, 5-, and 10-year limited duration prevalence was estimated as a cumulative incidence of cases diagnosed in the preceding 1, 5, and 10 years surviving to 2018. As an example, Fig. 3 shows estimation of 5-year prevalent cases of AL amyloidosis in 2018. Prevalence estimates were reported as PMP, calculated by dividing country-, age-, and gender-specific cases by United Nations’ country-, age-, and gender-specific population estimates [31].

## Results

### Incidence of AL amyloidosis

**Fig. 4:**
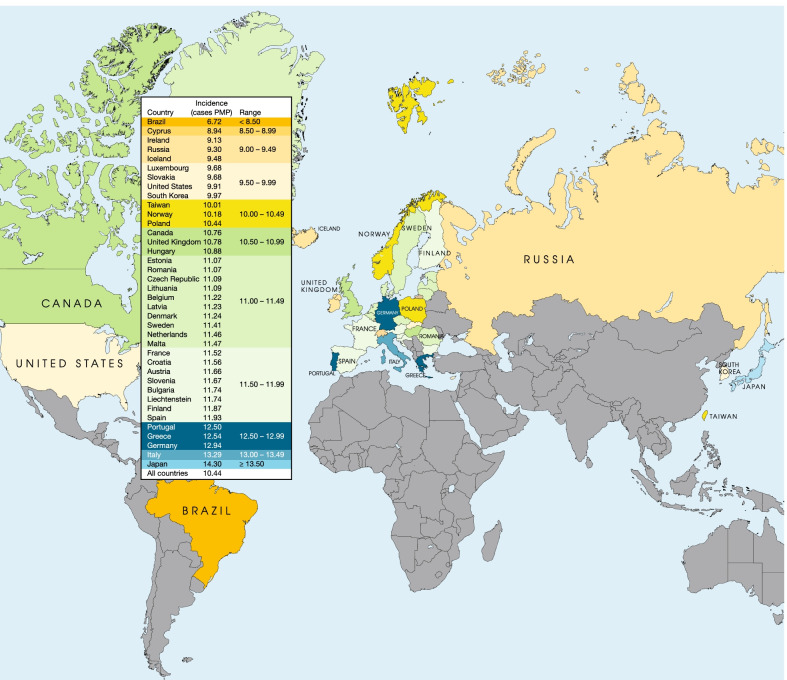
Calculated crude incidence in 2018, by country. *PMP *per million population

The estimated incident cases and incidence rates of AL amyloidosis in the countries under study are shown in Table 2 and Fig. 4. In 2018, a total of 14,982 incident cases was estimated for all countries. The crude annual incidence for all countries was estimated at 10.44 PMP, ranging from 6.72 PMP in Brazil to 14.3 PMP in Japan.

**Table 2:** Calculated crude incidence in 2018, by country

Country	Incident cases	Incidence (cases PMP)
Austria	100	11.66
Belgium	129	11.22
Bulgaria	83	11.74
Croatia	48	11.56
Cyprus	11	8.94
Czech Republic	117	11.09
Denmark	64	11.24
Estonia	14	11.07
Finland	66	11.87
France	751	11.52
Germany	1042	12.94
Greece	136	12.54
Hungary	106	10.88
Iceland	3	9.48
Ireland	44	9.13
Italy	795	13.29
Latvia	22	11.23
Liechtenstein	0	11.74
Lithuania	31	11.09
Luxembourg	6	9.68
Malta	5	11.47
Netherlands	196	11.46
Norway	55	10.18
Poland	402	10.44
Portugal	128	12.50
Romania	212	11.07
Slovakia	53	9.68
Slovenia	24	11.67
Spain	550	11.93
Sweden	114	11.41
United Kingdom	710	10.78
Brazil	1432	6.72
Canada	398	10.76
Japan	1798	14.30
Russia	1333	9.30
South Korea	508	9.97
Taiwan	236	10.01
United States	3260	9.91
All countries	14,982	10.44

*PMP* per million population

### Survival in AL amyloidosis

**Fig. 5:**
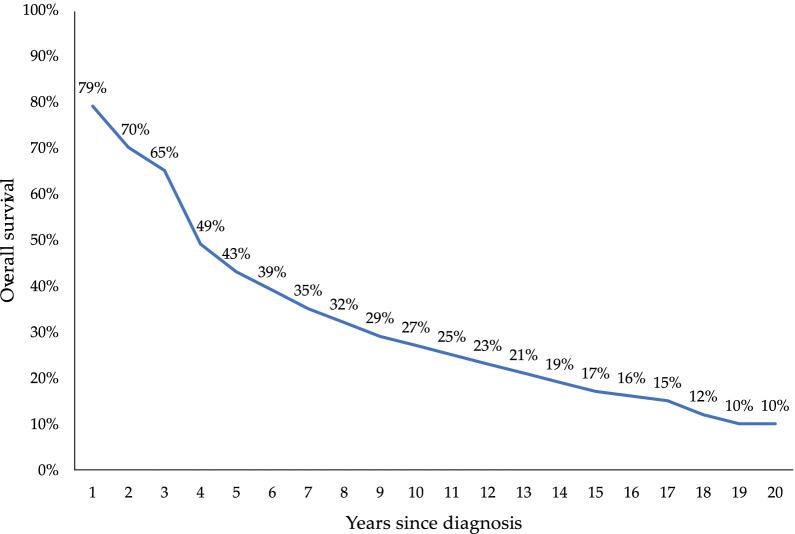
Relative survival of AL amyloidosis, by year since diagnosis. *AL* amyloid light-chain

The projected relative survival in AL amyloidosis for each year following diagnosis is shown in Fig. 5. In years 1, 5, 10, and 20 following diagnosis, relative survival was estimated at 79%, 43%, 27%, and 10%, respectively.

### Prevalence of AL amyloidosis

The estimated prevalent cases and prevalence of AL amyloidosis in the countries under study are shown in Table 3. In 2018, an estimated 73,567 cases were prevalent in all countries during the preceding 20 years. The 20-year prevalence for all countries was estimated at 51.27 PMP, ranging from 32.22 PMP in Brazil to 71.08 PMP in Japan. An increase in AL amyloidosis prevalence was observed over time in all the countries under study. One-year, 5-year, and 10-year period year prevalence of AL amyloidosis are also reported in Table 3.

**Table 3:** Calculated diagnosed prevalent cases and crude prevalence of AL amyloidosis since diagnosis, by country

	Prevalent cases				Prevalence (cases PMP)			
	1-year	5-year	10-year	20-year	1-year	5-year	10-year	20-year
Austria	79	284	406	497	9.12	32.92	47.14	57.70
Belgium	101	364	521	638	8.77	31.63	45.27	55.40
Bulgaria	64	234	337	415	9.15	33.28	47.90	58.92
Croatia	38	136	196	242	9.02	32.76	47.18	58.01
Cyprus	8	30	43	52	7.01	25.11	35.59	42.99
Czech Republic	92	331	473	577	8.67	31.32	44.81	54.62
Denmark	50	182	261	319	8.80	31.83	45.57	55.68
Estonia	11	41	59	72	8.63	31.25	44.88	55.10
Finland	52	187	267	327	9.29	33.60	48.10	58.85
France	586	2112	3025	3711	8.99	32.39	46.39	56.92
Germany	815	2950	4232	5203	10.11	36.62	52.53	64.59
Greece	106	385	552	676	9.78	35.40	50.74	62.19
Hungary	83	300	431	529	8.49	30.74	44.14	54.18
Iceland	3	9	13	16	7.43	26.74	38.17	46.43
Ireland	34	123	176	213	7.17	25.77	36.77	44.48
Italy	620	2235	3197	3909	10.37	37.37	53.48	65.38
Latvia	17	62	89	111	8.75	31.84	46.04	57.10
Liechtenstein	0	1	2	2	9.17	33.12	47.44	58.09
Lithuania	24	88	128	158	8.62	31.38	45.28	56.10
Luxembourg	4	16	23	28	7.58	27.23	38.57	46.53
Malta	4	14	20	24	9.00	32.56	46.58	56.41
Netherlands	153	554	793	969	8.98	32.45	46.44	56.72
Norway	43	154	220	268	7.97	28.67	40.85	49.77
Poland	314	1133	1619	1978	8.16	29.41	42.04	51.34
Portugal	100	362	521	636	9.76	35.41	50.93	62.16
Romania	165	599	861	1058	8.64	31.36	45.06	55.37
Slovakia	41	148	212	259	7.58	27.32	39.01	47.60
Slovenia	19	68	98	119	9.13	32.98	47.21	57.56
Spain	430	1551	2223	2704	9.32	33.62	48.20	58.64
Sweden	89	322	462	566	8.93	32.27	46.24	56.67
United Kingdom	555	2002	2863	3504	8.42	30.37	43.43	53.16
Brazil	1126	4018	5684	6859	5.29	18.88	26.70	32.22
Canada	312	1118	1589	1927	8.43	30.25	43.00	52.15
Japan	1402	5070	7279	8938	11.15	40.33	57.89	71.08
Russia	1040	3759	5380	6545	7.26	26.24	37.55	45.69
South Korea	399	1434	2034	2454	7.85	28.17	39.95	48.22
Taiwan	185	665	945	1142	7.86	28.24	40.13	48.52
United States	2552	9175	13,074	15,923	7.76	27.90	39.76	48.42
All countries	11,716	42,217	60,307	73,567	8.17	29.42	42.03	51.27

*PMP* per million population

## Discussion

This study used incidence and survival rates of AL amyloidosis identified through a SLR to estimate country-specific and global incidence and prevalence of AL amyloidosis. This analysis indicated that in 2018, the crude incidence of AL amyloidosis in countries in and near Europe, as well as in Brazil, Canada, Japan, Russia, South Korea, Taiwan, and the US was 10.44 PMP, with a prevalence of 51.27 PMP over the preceding 20 years. Orphan medicinal product designation criteria of the European Medicines Agency or Electronic Code of Federal Regulations indicate a disease must not affect > 5 in 10,000 people across the European Union or affect < 200,000 persons in the US [32, 33]. Our results elucidate the epidemiological impact of AL amyloidosis across the globe, indicating the rarity of this condition and confirming its orphan status (< 5 person per 10,000 in EU countries and < 200,000 total in the US, based on the current US population [34]). This study provides key information for initiatives focusing on extending lives and improving outcomes for patients with AL amyloidosis, such as through earlier diagnosis and the development of therapeutic interventions.

The present study showed small geographic variation in the estimates of AL amyloidosis incidence, which ranged between 8.94 to 13.29 PMP in countries in and near Europe and 6.72 to 14.3 PMP in Brazil, Canada, Japan, Russia, South Korea, Taiwan, and the US (Table 2, Fig. 4). Earlier studies conducted in France, Sweden and England reported lower estimates of AL amyloidosis. In France, a prospective study analyzing the epidemiology of AL amyloidosis between 2003 and 2005 estimated an incidence rate at 2.4 PMP [26]. In Sweden, a retrospective study extrapolating from myeloma data extracted from hospital discharge data between 2001 and 2008 estimated an incidence rate of 3.2 PMP [23]. In England, an epidemiological study that combined data for individuals referred to the NAC with death certificates between 2000 and 2008 estimated an incidence rate of 3 PMP [27]. The higher incidence rates of AL amyloidosis identified in this study may reflect increasing awareness of the disease and more efficient and earlier diagnosis in recent years [2, 35]. A study evaluating the trends in presentation, management, and outcomes among 1551 newly diagnosed patients with AL amyloidosis found patients diagnosed between 2010 and 2014 were less likely to have 2 or more affected organs than those diagnosed between 2000 and 2009, suggesting improved disease recognition before the onset of organ involvement [2]. In a review of records of 194 patients newly diagnosed with systemic AL amyloidosis between 2009 and 2016, a smaller percentage of patients were diagnosed with stage 4 disease compared to a large cohort of patients with long follow-up reported in 2012, suggesting that amyloidosis may be diagnosed earlier in the disease course in the present era [35]. Incidence of AL amyloidosis in the US estimated in this study was similar to that based on claims data from 2007–2015 and medical records from 1990–2015, where the incidence range was reported at 9.7–14.0 PMP in the US [3].

To the authors’ knowledge, no other studies have reported period prevalence of AL amyloidosis. In the present study, the prevalence of AL amyloidosis increased over the 20-year period of analysis from 8.17 PMP at 1 year following diagnosis to 51.27 PMP at year 20. Annual prevalence estimates reported previously were also found to increase over time [3, 27, 28]. In England, death certificates and referral rates to the NAC showed the estimated annual prevalence of AL amyloidosis in 2000 was 8.8 PMP, increasing to 20.4 PMP in 2008 [27]. In the US, claims data showed the estimated adjusted annual prevalence of AL amyloidosis increased significantly from 15.5 PMP in 2007 to 40.5 PMP in 2015 [3]. In Korea, data from the Korean National Health Insurance Service showed the overall age standardized annual prevalence of amyloidosis increased from an estimated 9.3 PMP in 2006 to 19.1 PMP in 2015 [28]. This apparent rise in the prevalence of AL amyloidosis may reflect an actual increase in disease incidence, improved survival resulting from recent advances in therapeutic options, increased disease awareness, and more appropriate diagnosis [2, 6, 35, 36]. In the US, OS among patients newly diagnosed with AL amyloidosis improved during 2010–2014 compared to 2000–2004 in association with a reduction in 6-month mortality and better response to therapy [2]. Similarly, in the US, a review of newly diagnosed patients with systemic AL amyloidosis conducted between 2009 and 2016 showed survival improvements for patients at all stages of disease [35].

### Limitations

The current study has several limitations. First, population-based studies to ascertain incidence and survival of AL amyloidosis are sparse and limited in geographical diversity limiting the generalizability of their findings to other populations. Second, derivation of AL amyloidosis incidence rates from a US-based study and extrapolation of survival rates from a Swedish experience to other countries may result in under- or over-estimation of incidence and prevalence of AL amyloidosis. Third, estimates of survival and incidence of AL amyloidosis from countries with well-developed healthcare systems may overestimate the number of prevalent cases in developing countries, where access to diagnosis and treatment is relatively limited.

Despite these limitations, this study fills a gap in the current understanding of the global epidemiology of AL amyloidosis by developing recent comparable epidemiologic data between the countries studied.

## Conclusion

This study provides up-to-date global estimates of the incidence and 1-year, 5-year, 10-year, and 20-year period prevalence of AL amyloidosis. Orphan medicinal product designation criteria of the European Medicines Agency or Electronic Code of Federal Regulations indicate a disease must not affect > 5 in 10,000 people across the European Union or affect < 200,000 people in the US. The epidemiological estimates for AL amyloidosis in this study highlight the rarity of condition across the globe and aid in understanding the true burden of the disease, which is vital for increasing awareness and furthering research and innovation of treatment options.

## Data Availability

No new data were created or analyzed in this study. Data sharing is not applicable to this article.

## Abbreviations

AL: Amyloid light-chain
FDA: Food and Drug Administration
NAC: National Amyloid Centre
ORR: Overall response rate
PI: Proteasome inhibitor
PMP: Per million population
PRISMA: Preferred Reporting Items for Systematic Reviews and Meta-Analyses
RRAL: Relapsed/refractory primary systemic AL Amyloidosis
SLR: Systematic literature review
US: United States
